# Ophthalmomyiasis Externa in an Atypical Region

**DOI:** 10.1155/crop/4330667

**Published:** 2026-04-25

**Authors:** Alyssa Snyder, Melissa Warne, Kyle Deistler, James Law, Carisa Bohnak, Edward Wladis

**Affiliations:** ^1^ Department of Ophthalmology, Lions Eye Institute, Albany, New York, USA, lei.org.au; ^2^ Department of Otolaryngology, Albany Medical Center, Albany, New York, USA, amc.edu

**Keywords:** botfly-related myiasis, climate change, ophthalmomyiasis externa, parasitic infection, pediatric ophthalmology

## Abstract

Ophthalmomyiasis is a rare condition caused by the infestation of ocular or periocular tissues by fly larvae. It is typically reported in tropical regions, rural settings, or among individuals with animal exposure. This report presents a unique case of ophthalmomyiasis externa in a 2‐year‐old girl from upstate New York. The patient presented with persistent left lower eyelid edema, erythema, and discharge following an insect bite that was unresponsive to antibiotic therapy. A botfly larva was expressed with digital pressure. The case underscores the diagnostic challenges of ophthalmomyiasis in unlikely regions and raises important questions about the ecological and environmental shifts facilitating the spread of parasitic diseases. Early recognition and intervention, including larval extraction and systemic therapy, proved effective. This report highlights the need for vigilance and broader differential diagnoses in regions where such infestations are uncommon.

## 1. Introduction

Ophthalmomyiasis is an uncommon parasitic condition characterized by the infestation of ocular or periocular tissues by fly larva. This pathological phenomenon manifests in two primary clinical forms. While some sources define ophthalmomyiasis externa narrowly as conjunctival involvement only, literature demonstrates considerable variation in this classification, including the invasion of external eye structures and adnexa, eyelid, conjunctiva, and lacrimal sac [1–5]. In contrast, ophthalmomyiasis interna affects intraocular structures. The *Oestrus ovis* larvae predominantly cause externa cases, while larvae of *Dermatobia hominis*, *Chrysoma bezziana*, *Hypoderma tarandi*, and *Cephenemyia trompe* are more frequently associated with interna manifestations [6].

The epidemiological landscape of ophthalmomyiasis is primarily associated with tropical and subtropical regions, particularly among individuals with extensive travel to endemic areas, occupational exposure to livestock, or close contact with specific animal populations [6–8]. Isolated cases in temperate regions remain exceedingly rare. However, rare previous reports have demonstrated the potential for ophthalmomyiasis in unexpected geographical contexts, including Nearctic regions and suburban environments [9, 10].

Recent observations suggest that environmental and ecological factors contribute to the expansion of parasitic disease transmission for conditions such as ophthalmomyiasis. Global environmental changes, including climate transformation and shifts in arthropod vector habitats, enable traditionally tropical species to extend their geographic range [11]. Furthermore, increased globalization and international trade facilitate the inadvertent transportation of parasitic organisms to non‐endemic areas [12]. Diagnosing ophthalmomyiasis in temperate climates is challenging due to its rarity and clinical resemblance to more common conditions. However, prompt and accurate diagnosis is crucial, as delays or misdiagnoses may result in prolonged symptoms or complications [6]. Treatment approaches are diverse, including mechanical larval extraction, suffocation techniques, and systemic medical management [6, 13–15].

This report presents a unique case of ophthalmomyiasis externa in a pediatric patient from New York State. By examining this case’s clinical and epidemiological context, we aim to raise awareness of ophthalmomyiasis diagnosis, investigate potential mechanisms of parasitic disease transmission, and emphasize the need for heightened diagnostic vigilance, even in regions traditionally considered low‐risk.

Informed consent was obtained from the patient’s legal guardian for the publication of this case report including identifiable photographs. The collection and evaluation of protected patient health information were conducted in compliance with HIPAA regulations and in accordance with the tenets of the Declaration of Helsinki.

## 2. Case Presentation

A 2‐year‐old girl from upstate New York without significant medical history developed swelling and discharge of the left lower eyelid after an insect bite. Despite initial treatments with oral cephalexin and trimethoprim‐sulfamethoxazole, the lesion continued to worsen, and the patient started experiencing nausea, vomiting, and malaise, which prompted presentation to the emergency department.

**Figure 1:**
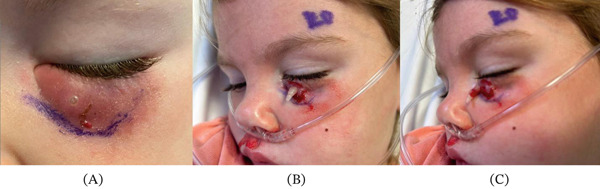
(A–C) Mechanical larval extraction progression. (A) Initial presentation and positioning of the larva prior to extraction. (B) Application of pressure and mechanical removal of the larva. (C) Successful extraction demonstrating complete larval removal.

On exam, the patient was afebrile. Her left lower eyelid exhibited moderate erythema and edema, with a 1 mm open wound that was intermittently draining serous fluid (Figure 1A). The eye was otherwise white and quiet. The patient was able to fix and follow a target at 8 feet, with no afferent pupillary defect. Intraocular pressure was normal, and extraocular movements were full. Ultrasound showed no discrete fluid collection in the orbit, though indeterminate calcifications were noted.

Application of pressure surrounding the open wound was performed, and a larva was found and removed from the eyelid (Figure 1B,C). The patient was given a dose of ivermectin and underwent computed tomography (CT) of the head, which was negative for further parasitic burden. She was discharged on oral ivermectin with complete recovery. The larva was confirmed to be a botfly by an arthropod expert at the New York Department of Health, though species identification was inconclusive.

## 3. Discussion

Ophthalmomyiasis is a rare but notable condition caused by the infestation of tissues by fly larvae surrounding or within the eye [6]. It is most reported in people working with animals and farming, but is also commonly seen in tropical and subtropical rural areas, where hygiene is poor and flies are abundant [7, 8]. However, this case is particularly notable due to its diagnosis in New York, a region traditionally considered low‐risk for ophthalmomyiasis. The occurrence of ophthalmomyiasis externa in this geographic area introduces new challenges and underscores the potential impact of environmental and ecological changes, including climate change, as well as increased human–insect interactions in non‐traditional settings.

The diagnosis of botfly‐related ophthalmomyiasis is considered exceedingly rare in temperate regions like New York, with only one previously reported case of botfly‐related respiratory myiasis in this region [16]. While typically endemic to neotropical regions of Central and South America and often associated with international travel [13, 14, 17, 18], cases have also been documented in India and the Middle East [19, 20], as well as unexpected locations such as northern Canada and urban areas in France [9, 10].

Botfly species native to the northeastern United States typically do not infect humans, and literature on isolated cases remains limited [21–23]. It is unclear whether this patient’s infestation resulted from exposure to a native species or the introduction of a non‐native species to the region. These reports indicate the potential for human infestation in colder climates and underscore the impact of urbanization and ecological changes, which may increase human exposure to insects capable of causing myiasis. Clinicians should remain vigilant and consider ophthalmomyiasis in the differential diagnosis of persistent ocular adnexal infection, even in regions far removed from typical botfly habitats and parasitic infections.

This unusual presentation raises important questions about potential contributing factors. It may reflect changing environmental and ecological conditions, such as global warming, which could enable tropical species to expand their range into temperate regions like New York. Increased temperatures through climate change have shown to aid in the development of arthropod vectors that may carry parasitic organisms, increasing overall parasitic transmission, and altering their spread and distribution [11]. Alternatively, it could result from human activities and globalization, such as importation of goods or animals, that inadvertently transport botfly eggs or larvae to non‐endemic areas, creating isolated opportunities for infestation [12].

Because of its regional rarity, opthalmomyasis externa diagnosis can be complicated and mistaken for more common periocular conditions such as viral or bacterial conjunctivitis, or preseptal or orbital cellulitis [6]. The larval spines create punctate hemorrhages due to microtrauma and appear very similar to those seen in adenoviral conjunctivitis [6]. Examination of the fornices and slit lamp exam is required, as some larvae tend to hide there [5]. Given the rarity of ophthalmomyiasis, healthcare providers unfamiliar with the condition may fail to consider it as a differential diagnosis when presented with symptoms resembling more common eye infections.

Several methods of treatment have been reported for ophthalmomyasis externa. Although the condition can be self‐limiting, early resolution of symptoms is often desired. The primary method of treatment involves the mechanical extraction of the larvae [6]. In cases where extraction is challenging due to the depth of invasion or the location of the larvae, various alternative methods have been documented. These include suffocation techniques using agents such as pork fat, raw meat, petroleum jelly, liquid paraffin, nail polish, adhesive tape, chewing gum, bee’s wax, and different oils [13, 14]. These substances can effectively bring the larvae to the surface, triggering their search for air, thereby facilitating extraction.

Additionally, systemic treatment with oral ivermectin has been found to be both safe and effective in managing ophthalmomyiasis [13, 15]. In the case of our patient, an oral dose of ivermectin was administered to ensure the elimination of any remaining parasitic burden and to promote a faster recovery. This treatment strategy was chosen to complement the mechanical extraction and provide a more comprehensive approach to managing the condition.

This case of ophthalmomyiasis externa highlights the evolution of parasitic infections in unforeseen areas due to environmental, ecological, and human factors. The unusual location of the diagnosis raises important questions about the influence of climate change and human activity in facilitating the spread of tropical species to temperate regions. Given the rarity and clinical resemblance to more common eye conditions, ophthalmomyiasis must remain a consideration in differential diagnoses, particularly in regions where such cases have not been previously reported.

## Author Contributions

Alyssa Snyder, BS – data collection, management, and interpretation, literature review, writing and editing, project administration.

Melissa Warne, MD – data collection, management, and interpretation, writing and editing, critical review.

Kyle Deistler, MD – study concept and design, writing and editing, critical review.

James Law, MD – study concept and design, writing and editing, critical review.

Carisa Bohnak, MD – study concept and design, writing and editing, critical review.

Edward Wladis, MD – study concept and design, writing and editing, critical review supervision and oversight, project administration.

## Funding

No funding was received for this manuscript.

## Disclosure

All authors have reviewed the final version to be published and agreed to be accountable for all aspects of the work.

## Ethics Statement

Albany Medical Center determined that ethical approval for reporting individual cases was not required. Our IRB determined that the case report was not human research and was exempt (Rapid IRB Determination #: R_3aqqsMrZU4BTYHg).

## Conflicts of Interest

The authors declare no conflicts of interest.

## Data Availability

Data sharing is not applicable to this article as no new data were created or analyzed in this study.
